# Estimating the cost-effectiveness of the National Weight Management Enhanced Service and NHS Digital Weight Management Programme in England: a modelling study

**DOI:** 10.1136/bmjopen-2026-117143

**Published:** 2026-10-12

**Authors:** Ben Amies-Cull, Stella Haffner, Peter Scarborough, Sarah Mounsey, Paul Aveyard, Susan Jebb

**Affiliations:** 1 Nuffield Department of Primary Care Health Sciences, University of Oxford, Oxford, UK; 2 NIHR Oxford Health Biomedical Research Centre, Oxford, UK; 3 Department of Neuroscience, University of Copenhagen, Copenhagen, Denmark; 4 MRC Epidemiology Unit, University of Cambridge, Cambridge, UK; 5 NIHR Oxford Biomedical Research Centre, Oxford, UK; 6 NIHR ARC Oxford and Thames Valley, Oxford, UK

**Keywords:** Preventive Health Services, Obesity, HEALTH ECONOMICS, Digital Technology

## Abstract

**Abstract:**

**Objectives:**

To assess the long-term cost-effectiveness of the National Health Service (NHS) Digital Weight Management Programme.

**Design:**

Administrative data analysis and modelled cost-effectiveness.

**Setting:**

NHS in England.

**Participants:**

A total of 31 753 participants (20 379 females and 11 374 males) completed the programme.

**Intervention:**

In March 2021, NHS England initiated a National Enhanced Service incentive for General Practitioners (GPs) to refer patients with obesity plus diabetes and/or hypertension to a 12-week behavioural weight management intervention, the NHS Digital Weight Management.

**Primary and secondary outcomes:**

Cost-effectiveness as cost/quality-adjusted life-years (QALYs). The PRIMEtime model (a population model) was used to estimate long-term impacts of this weight loss on QALYs, costs to the NHS and domiciliary social care over participants’ lifetimes, discounted at 3.5% for health and costs and cases of 20 non-communicable diseases over 30 years. Weight loss was estimated in 5-year age and sex groups for completing participants.

**Results:**

Mean weight loss was 1.18 kg/m^2^ (95% CI 1.15 to 1.21) for males and 1.20 kg/m^2^ (1.18 to 1.22) for females. Over the lifetime, 0.0958 QALYs (0.0746–0.126) were saved per male and 0.110 (0.864–0.146) per female, with £65.0 (£31.8–111) and £170 (£112–248) costs to the NHS, respectively, and £50.9 (£20.7–72.0) and £229 (£172–300) to social care. Intervention costs were £133.49 per completer, giving a cost per QALY of £4202 (£3437–5064) from a health and social care perspective.

**Conclusions:**

The programme was highly cost-effective to national standards, robust to probabilistic sensitivity analysis and a range of deterministic sensitivity analyses on weight regain, counterfactual weight loss and discount rates.

**STRENGTHS AND LIMITATIONS OF THIS STUDY**

- This study used a large routinely collected dataset and advanced simulation modelling methods to estimate long-term health impacts of the NHS Digital Weight Management Programme.
- It incorporates detailed cost data to allow cost-effectiveness of the programme to be estimated, though some of these costs were commercially sensitive so not available.
- The study design of the effect of the programme relies on observational data, and the counterfactual was estimated based on previous studies.
- It includes extensive sensitivity analyses that support the findings of the primary analysis.

## Introduction

 Behavioural weight management is known to reduce obesity-related health risk. In response to the COVID-19 pandemic and the evidence that people with obesity were at increased risk,^1^ the government in England stepped up action to support people living with obesity to lose weight. In April 2021 a national programme offering access to the NHS Digital Weight Management Programme was made available and GPs were financially rewarded as part of a National Enhanced Service (NES) to refer people living with obesity and either diabetes or hypertension to a weight management programme.^2^ We have previously shown that behavioural weight management programmes would be cost‐effective, from a health service perspective, if delivered for under £560 (£8.80–3,900) per person.^3^ Yet referral to weight management programmes is still not commonplace in England, in common with most other parts of the world.

The programme is provided as 10 contracted services by six commercial providers as a free-to-user 12-week weight management intervention. To increase participant retention, patients referred to the programme are triaged to different levels of programme support depending on various demographic factors, which have been shown to influence programme engagement and retention (previously described in Taylor *et al*^2^). Level 1 provides self-guided digital content, alongside optional participation in group peer-support sessions; Level 2 additionally offers up to 50 min of one-to-one coaching by video call, phone or in app messaging/contact over the 12 weeks; and Level 3 provides 100 min of this coaching. The programme incentivises patient retention by paying providers only for users who completed at least 7 of the 12 weeks of the programme.

Flow through the system starts with a referral from the GP. The patient is then triaged to different levels of support and the patient can choose which of the six commercial providers they prefer. They are then contacted by that provider and can choose whether to take up the offer of support. A flow diagram to demonstrate this is included in online supplemental figure 1.

In the first year of the programme, 45% of participants who were triaged to a provider completed the programme, with mean weight loss of −3.9 kg (95% CI −3.84 to −3.99) across the three levels.

2 years after the launch of the NES, it is now possible to answer more detailed questions about the NES and the NHS Digital Weight Management Programme. In this study, we aimed to assess:

1. The uptake, completion and weight loss achieved by participants referred under the NES in the programme 2021–2023.
2. The modelled long-term effects of these programme participants on health outcomes, healthcare and social care costs for those who completed the programme.
3. The cost effectiveness of the NES incentive and referral to programme from a health and social care perspective.

## Methods

### Data

The data were compiled by NHSE using referral information from participants’ GPs and programme data from service providers. The dataset included demographics, eligibility criteria and relevant comorbidities at point of referral. Providers populated a minimum dataset that included individual participants’ self-reported weight measurements and weekly engagement with the provider programme.

### Uptake, completion and weight change

Programme uptake is defined as the proportion of referred patients that have chosen a provider, recorded a programme start date and engaged in at least 1 week with the provider programme. Those who complete the programme are defined as those who had time to finish and engaged in 7 weeks out of the 12. Engagement with the provider programme in a given week is defined as at least one of the following forms of engagement: self-recording a weight measurement, receipt of human coaching and/or engagement with the provider, all recorded digitally. Weight change was taken from self-reported weight in the latest week recorded minus that in the earliest (including the GP referral if two were not available in the programme), and Body Mass Index (BMI) changes calculated using the height reported in the GP referral. Outlier BMI starting values and change values >5 SD from the mean were treated as non-valid and excluded. Mean BMI changes for health modelling were calculated by 5-year age/sex groups. Participants without two reported BMI values and outliers were assumed to have had no weight change. This approach is slightly different from that taken in Taylor *et al*,^2^ particularly in that those without valid weight measurements were excluded from the estimate of mean weight change in the previous paper but included as zero change here, to avoid biasing up the intervention effect while maintaining the correct number of participants who completed the intervention.

### Modelled outcomes

Mean BMI change among those who completed the programme by age and sex was modelled through to long-term health outcomes, healthcare and social care costs using the PRIMEtime model. This has been described in detail elsewhere.^4 5^ Briefly, PRIMEtime is a proportional multistate lifetable model,^6^ which simulates long-term outcomes of an exposure to a risk factor, twice in parallel: once at baseline and once with a scenario alteration to risk factor exposure applied. Disease outcomes are mutually independent of each other with the exception of type-2 diabetes, as this confers additional risk for Ischaemic Heart Disease (IHD) and stroke. The prevalence of type-2 diabetes in this population was much higher than the general population, so this was accounted for separately. Details of the method used to account for increased diabetes risk are described in the supplementary material and online supplemental table S1. In short, this relied on a separate run of the Disbayes model^7^ (a Bayesian epidemiological model) to generate coherent incidence, prevalence and mortality estimates for Type-2 Diabetes Mellitus (T2DM) in our population. For this run, we used prevalence estimates from the NHS England data on our cohort. The incidence and mortality rates were estimated in a separate version of PRIMEtime, which was set up using data from the general population and which estimated the T2DM incidence and mortality impact of an increase in BMI to match the distribution seen in our NHS England cohort.

Here, PRIMEtime estimated the impact on the number of cases of 20 BMI-related diseases: ischaemic heart disease, ischaemic stroke, intracranial haemorrhage, hypertensive heart disease, type 2 diabetes, colorectal cancer, breast cancer, uterine cancer, oesophageal cancer, kidney cancer, pancreatic cancer, liver cancer, multiple myeloma, asthma, low back pain, osteoarthritis of the hip, osteoarthritis of the knee, depression, atrial fibrillation/flutter and gallbladder/biliary diseases. The input epidemiology of these diseases is given in online supplemental figures 1–5. Change to disease incidence rates are estimated using the population impact fraction, that is, the proportion of a disease’s burden at baseline that is attributable to the change to risk factor being modelled in the scenario. The relative risks for these relationships are taken from observational studies and given in online supplemental tables S2 and S3.

People in the baseline and scenario simulations were assumed to follow a path of slowly rising body weight with age. The intervention involved a reduction in weight at the point of the programme, while the comparator remained on the age-related path. After the end of the programme, we assumed that participant weight would be slowly regained at 0.33 kg/year (95% CI 0.24 to 0.43) following findings of a systematic review of long-term outcomes of behavioural weight loss interventions^3^ until body weight matched that of the base case. The age, sex, and starting BMIs of participants completing the programme were used as baseline characteristics in the model, and the simulation was run from 2022 over the remaining natural lifetime of the participants by applying age- and sex-specific all-cause mortality rates, to a maximum age of 100 years for quality-adjusted life-years (QALYs), healthcare and social care costs. For disease cases, participants were simulated through remaining lifetime over a maximum 30-year horizon.

UK utility decrements (Sullivan *et al*,^8^ given in online supplemental tables S4 and S5) were used to calculate incremental QALYs. Healthcare costs were included per year or as one-off treatment costs, depending on disease (online supplemental table S6), and social care costs were modelled via the mean hours of formal domiciliary care received, by BMI value.^9^ The mean cost per hour of formal care was taken as £22.55 from Department of Health and Social Care data.^10^ Baseline BMI distributions were smoothed, taking the predicted values from a linear model, with independent terms age*sex and age2, based on model output Wald tests. Baseline population and BMI distributions are given in online supplemental tables S7 and S8, respectively.

### Health economic evaluation

The total programme costs include (i) an £11.50 incentive payment to GPs per referral, (ii) payment to a centre for hub services (the NHS Midlands and Lancashire Commissioning Support Unit) for each patient who contacted the triage hub to be allocated to an intervention level and select a provider and (iii) payment to the weight management programme providers for each patient who completed the programme (providers were not paid for participants who did not engage for 7 of the 12 weeks). Programme provider fees varied according to the level of support. Due to commercial sensitivity, it is not possible to share disaggregated figures for providers or the triage service. Impacts on healthcare costs, social care costs and QALYs were taken from PRIMEtime outputs.

Modelled health and cost outcomes were discounted to 3.5%, and cost-effectiveness presented as cost per QALY and as Net Monetary Benefit (NMB) per person, in line with guidance from the National Institute for Health and Care Excellence (NICE) (PMG36). NMB was calculated as: QALY benefits × Willingness To Pay threshold, minus cost impacts of all people referred to the programme. The cost impacts were for all patients and all sources of costs, including all referral costs, even if a patient was not then triaged, or triage costs even if a patient did not engage with the provider, and provider costs, even if a participant did not lose any weight, as these were the costs incurred by the NHS. This used a Willingness To Pay threshold of £20 000/QALY, in line with NICE guidance, divided by the number of completers.

### Sensitivity analyses

Six sensitivity analyses were performed.

1. Probabilistic sensitivity analysis was conducted by 2000 iterations of Monte Carlo analysis in PRIMEtime. Parameter uncertainty was included for disease relative risks, utility decrements, healthcare costs, social care costs, the intervention effect (BMI change) and weight regain trajectory.
2. Social care costs were excluded to estimate cost-effectiveness from a pure NHS perspective.
3. The cost per QALY of the programme alone, including triage costs but excluding the NES incentive, from an NHS and social care perspective.
4. An alternative counterfactual was implemented where participants lost 1 kg, based on a meta-regression of control groups in behavioural weight loss trials.^11^ This weight loss was assumed to be regained at the same rate as that under the intervention.
5. The weight lost in the programme was regained at 0.5 and 1.5 times the value in the main analysis.
6. Alternative discount rates were used: 1.5% for health and costs, at Treasury rates (for health outcomes 1.5% for years 1–30, 1.29% for years 31–75 and 1.07 for years 75–100, or 3.5%, 3% and 2.5% for costs, respectively), and undiscounted.
7. Supplementary sensitivity analysis 1: complete case analysis, excluding the 161 patients with missing BMI change data, rather than assuming 0 kg intervention weight impact.
8. Supplementary sensitivity analysis 2: assuming a counterfactual weight loss of 2 kg.
9. Supplementary sensitivity analysis 3: assuming a rate of weight regain at twice that of the main analysis.

## Results

### Study participants

Between 12 April 2021 and 31 March 2023, 202 355 people were referred by their GP to the NHS Digital Weight Management Programme. Of these, 134 346 patients were triaged and selected a provider (66.4%), 68 938 people started and engaged in at least 1 week of the programme, and 31 753 completed it: 17 126 in Level 1, 7732 in Level 2 and 6895 in Level 3. The uptake was therefore 34.1% of those who selected a provider, and completion was 46.1% of those who engaged at least once, or 15.7% of those referred. These 31 753 participants make up this study’s sample for further analyses and health modelling, and further baseline and demographic data are given in table 1. Of these, 161 participants were assumed to have zero weight loss, due to not having two weight measurements or having outlying baseline BMI or BMI change values. Given the nature of the data from the NHS record and commercial provider referrals, there was very little missing data, with 65 missing from the sex variable, none from the age variable or starting BMI. None of the 65 participants with missing sex values completed the programme, so were all excluded from analysis.

**Table 1:** Baseline characteristics of study participants completing the NHS Digital Weight Management Programme

Characteristic	Males	Females	Total
Mean age (years) (SD)	56.2 (12.1)	54.9 (12.1)	–
Gender (%)	11 374 (35.8)	20 379 (64.2)	31 753
Ethnicity (%)			
Asian	1439 (12.7)	2285 (11.2)	3724 (11.7)
Black	633 (5.57)	1981 (9.72)	2614 (8.2)
Mixed	174 (1.53)	421 (2.07)	595 (1.9)
Other	589 (5.18)	1090 (5.35)	1679 (5.3)
White	8539 (75.1)	14 600 (71.6)	23 139 (72.8)
Unknown		2 (0.00981)	2 (0.01)
Deprivation quintile* (%)			
1 (most deprived)	2272 (20)	4946 (24.3)	7218 (22.7)
2	2254 (19.8)	4377 (21.5)	6631 (20.9)
3	2340 (20.6)	4055 (19.9)	6395 (20.1)
4	2349 (20.7)	3857 (18.9)	6206 (19.5)
5	2159 (19.0)	3144 (15.4)	5303 (16.7)
Intervention level (%)			
Level 1	6359 (55.9)	10 767 (52.8)	17 126 (53.9)
Level 2	2686 (23.6)	5046 (24.8)	7732 (24.3)
Level 3	2329 (20.5)	4566 (22.4)	6895 (21.7)
Has hypertension only (%)	5079 (44.7)	10 391 (51)	15 470 (48.7)
Has diabetes only (%)	3041 (26.7)	5722 (28.1)	8763 (27.6)
Has diabetes and hypertension	3254 (28.6)	4258 (20.9)	7512 (23.6)
Median starting BMI (kg/m²) (IQR)	35.1 (7.65)	36.8 (8.22)	

* Postcode-level quintile of English Index of Multiple Deprivation (IMD) 2019. IMD is a measure of relative disadvantage with quintile 1 being the most disadvantaged and quintile 5 the least.

BMI, Body Mass Index; NHS, National Health Service; SD, Standard Deviation.

### Weight and BMI change

Overall mean weight loss for people completing the programme was 3.68 kg (95% CI 3.60 to 3.76 kg) for males and 3.17 kg (95% CI 3.12 to 3.22 kg) for females, equivalent to a reduction in BMI of 1.18 (1.15–1.21) and 1.20 (1.18–1.22) kg/m^2^, respectively. Weight changes by 5-year age and sex group (used for health modelling) are shown in figure 1.

**Figure 1:**
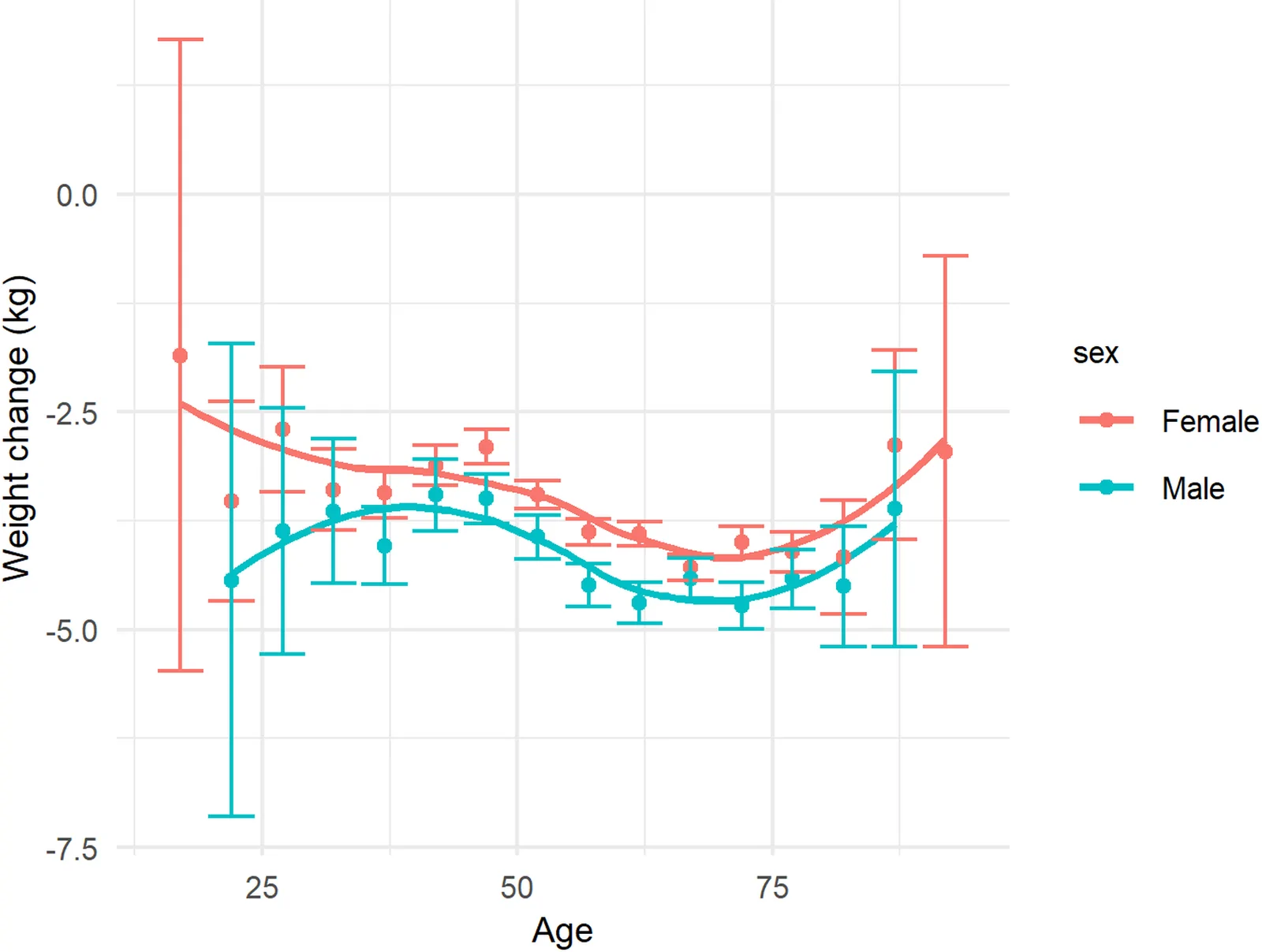
5-year age and sex group average body weight changes achieved in the NHS Digital Weight Management Programme (points) with smoothed average (lines, by GAM regression). GAM: Generalised Additive Regression; kg: kilogram; NHS: National Health Service.

### Modelled outcomes

Table 2 summarises the modelled impacts of the programme on disease cases, QALYs, healthcare and social care costs.

**Table 2:** Modelled impact of the NHS Digital Weight Management Programme on health and cost outcomes

	Males			Females		
	Mean	Lower 95% UI	Upper 95% UI	Mean	Lower 95% UI	Upper 95% UI
Per person over 100 years						
QALYs	0.0958	0.0746	0.126	0.110	0.086	0.146
NHS costs	65.0	31.8	111	170	112	248
Domiciliary SC costs	50.9	20.7	72.0	229	172	300
Cases per 10 000 at 30 years						
Asthma	2.38	1.35	3.63	8.41	6.32	11.1
Gallbladder diseases	−147	−222	−96.9	−176	−264	−117
Depressive disorders	80.3	62.7	104	139	110	182
Low back pain	42.9	19.7	70.5	103	73.5	142
Osteoarthritis hip	−0.336	−1.19	0.476	0.0749	−0.882	1.03
Osteoarthritis knee	6.74	4.31	9.47	16.3	12.3	21.1
Ischaemic heart disease	−33.2	−51.0	−22.4	−10.4	−17.9	−5.98
Ischaemic stroke	−4.12	−6.98	−2.28	−1.97	−4.30	−0.510
Intracerebral haemorrhage	−1.35	−2.62	−0.35	−0.149	−0.950	0.477
Hypertensive heart disease	−2.46	−4.80	−0.908	−1.04	−2.31	−0.101
Diabetes mellitus type 2	−275	−375	−210	−216	−300	−165
Atrial fibrillation and flutter	−8.80	−14.7	−4.08	−3.97	−7.98	−0.790
Colorectal cancer	3.39	2.48	4.46	3.25	2.51	4.17
Breast cancer	–	–	–	2.71	1.51	3.91
Uterine cancer	–	–	–	−1.55	−2.35	−1.06
Oesophageal cancer	−2.83	−4.56	−1.64	−0.864	−1.56	−0.424
Kidney cancer	−0.483	−1.05	−0.0583	−0.104	−0.383	0.106
Pancreatic cancer	0.515	0.131	0.905	0.834	0.505	1.22
Multiple myeloma	0.154	0.0135	0.288	0.221	0.132	0.314
Liver cancer	−0.679	−1.50	−0.0212	−0.210	−0.629	0.133

NHS, National Health Service; QALYs, quality-adjusted life-years; SC, Social Care; UI, Uncertainty Interval.

#### Disease cases

Reductions in incident cases of type-2 diabetes of 275 cases/10 000 males (95% UI −210 to −375) and 210 cases/10 000 females (−165 to −300) were modelled with all-cause mortality applied up to a maximum of 30 years. We predicted modest reductions to incident cases of ischaemic heart diseases (−33.2 cases/10 000 males (−22.4 to −51.0) and −10.4 cases/10 000 females (−5.98 to −17.9)). Likewise, modest impacts were modelled on incident cases for cancers, mostly to slightly increase numbers overall. The greatest impact was on colorectal cancer, with an increase of 3.39 cases/10 000 males (2.48–4.46) and 3.25 cases/10 000 females (2.51–4.17). Incident cases of gallbladder and biliary diseases showed a reduction (of 147 cases/10 000 males in 30 years (96.9–222 and 176 cases/10 000 females, 117–264). Cases of low back pain were estimated to increase by 146 cases/10 000 participants (93.2–213).

#### Costs and QALYs

The programme increased total NHS disease treatment costs by £4.20 m (95% UI £2.64 m–£6.33 m) and increased social care costs by £5.25 m (95% UI £3.74 m–£6.94 m) over completers’ remaining lifetime (100 years). These are given in table 2 per 10 000 participants (by sex) over 30 years. Females are estimated to incur much greater impacts on NHS costs and domiciliary care costs than males, due to greater longevity.

### Health economic evaluation

The mean cost of provider fees plus triage costs was £60.20 per completer, including triage costs for all 202 355 referred patients, including dead-weight costs for those who did not engage. Provider fees varied by intervention level (numbers above, under *Study participants*), paid for each of 31 753 participants completing the programme. Including GP referral fees, the total cost per completer was £133.49. Estimated net cost/QALY for the NES and the programme was £4202/QALY (95% UI £3437–£5064). NMB at £20 000/QALY was £1,530 (£1120–£2140) per completer.

#### Sensitivity analyses

Results for the probabilistic sensitivity analysis are given in figure 2. This demonstrates that the intervention remains overall cost-effective for all primary and sensitivity analyses. Cost per QALY for NHS costs alone (excluding social care) was £2596 (95% UI £2088–£3138). Including both NHS and social care costs but excluding referral costs, it would be £3499 (£2854–£4233). Using a counterfactual baseline population with a weight loss of 1 kg had a cost per QALY of £4323 (£3502–£5129). When the rate of regain was assumed to be 0.5 times that in the primary analysis it was £3031 (£2232–£3677), and for 1.5 times the rate of regain it was £4857 (£3981–£5657). Undiscounted, the cost/QALY was £5434/QALY (£4630–£6328), for 1.5% discount rates on health and costs £4854 (£4009–£5619), and for Treasury rates £2928 (£2368–£3463). Additional sensitivity analyses extending these are reported in the online supplemental material.

**Figure 2:**
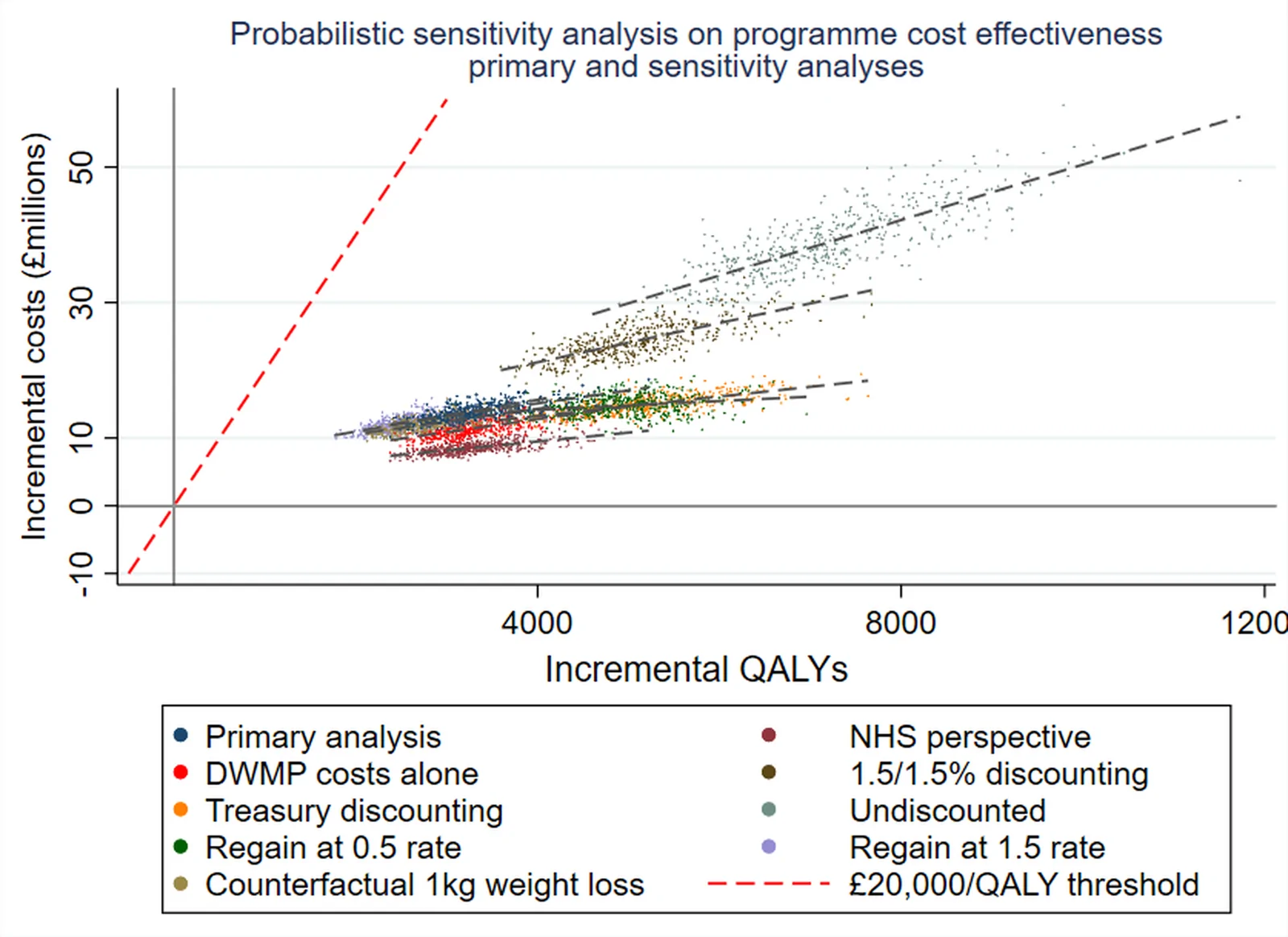
Cost-effectiveness plane showing probabilistic sensitivity analyses on primary and sensitivity analyses, with £20 000/QALY threshold in red dashed line. QALY, quality-adjusted life-years; NHS, National Health Service; DWMP, Digital Weight Management Programme.

## Discussion

This analysis estimates that the NES incentive with referral to the NHS Digital Weight Management Programme to be highly cost-effective. Of those referred, 51.5% signed up to a provider and 30.4% of these participants completed the programme with an average weight loss of 3.35 kg. When modelled over participants’ remaining lifetime this programme was estimated to save 1.06 QALYs per completer, with a net cost to the NHS of £234 m and a further £281 m to domiciliary social care, due to increased longevity. The cost-effectiveness from a health and social care perspective was £4202/QALY. These estimates include the full costs of administering and delivering the programme, including all referral and triage fees for those who did not attend or complete the programme.

### Strengths and limitations

This is the first evaluation of the cost-effectiveness of a national incentive for weight management combined with a digital weight management service. We obtained detailed information on the costs of the service, its participants and results from NHS England. In a wide variety of deterministic sensitivity analyses, alongside probabilistic sensitivity analysis, the impact on our primary findings was modest and did not alter the interpretation that this system of care was highly cost-effective.

Due to this being unblinded observational data, the risk of reporting bias cannot be excluded. These self-reported data may be open to an optimism or desirability bias of losing more weight during the intervention. However, the amount of weight loss was similar to what we might expect and sensitivity analyses show modest impacts on the interpretation of programme cost-effectiveness. Outliers with weight changes >5 SD from the mean were excluded as a small number of cases reported extreme values that were not credible in a 12-week weight intervention, such as reporting over 100 kg of weight loss (n=24) or even 100 kg weight gain (n=11) that were most likely due to user error. The intervention population was not nationally representative, for example having nearly double the number of females completing as males, highlighting a potential limitation of the intervention. However, this was complete real administrative data so was therefore representative of participants for this kind of intervention (eg. greater female participation is expected), this should not bias the cost-effectiveness result. Outcomes for the 16% who completed the programme may not be representative of the general population, however as non-completers did not trigger provider payment fees, this did not bias this cost-effectiveness analysis. PRIMEtime was developed as a population model to represent the average risks across the population of England. We were able to adapt it to account for increased baseline risks associated with this patient group’s high rates of type-2 diabetes, which were reported in the NHS England data, though we could not account for the possibility of diabetes remission. This was likely to be low given the modest weight loss and likely regain over time. Accounting for hypertension-related risk fell outside the scope of the project due to data limitations limiting the ability to appropriately capture this risk. This was due to participants being classified as a binary hypertensive/not hypertensive rather than having a continuous blood pressure measurement in mm Hg.

The choice of counterfactual in the health modelling is taken to be no weight loss, though it is possible that participants who are offered a referral to a programme might be motivated to undertake increased personal efforts to manage their weight even if they do not attend the prescribed programme. We have previously shown that following opportunistic advice to recommend weight loss, 79% of people reported taking action over the following 12 weeks with a mean weight loss across the whole group of 1.18 kg.^12^ The sensitivity analysis exploring this found a modest reduction in the cost-effectiveness of the intervention, as would be expected, but still representing good value for money to the NHS. Wider social and economic costs were not included in this analysis, such as absenteeism, disability-related unemployment, or working age mortality. These would be likely to enhance the cost-effectiveness, though there is also potential for the intervention to increase pension costs. Informal social care hours are also not included, which are likely to be a considerable social benefit, given the strength of association between BMI and informal care.^9^

### Comparison with previous work

Early outcomes of this programme have been previously reported,^2^ including a 50% uptake and 45% completion rate. Weight change is not directly comparable between the two papers due to differences in methods. Despite this success, the proportion of patients referred who complete the programme was only around 16% and further work is needed to boost uptake and adherence.

To our knowledge, this is the first long-term economic analysis of a digital behavioural weight management intervention. Examining sources of costs in the overall pathway, the triage and the programme components were shown to be very cost-effective, not least because payments to providers were contingent on completion of the programme. Other routes into the programme, for example, self-referral, would be likely to attract a different population and weight loss may vary, with potentially different cost-effectiveness.

### Implications for policy and practice

Obesity is a known risk factor for chronic disease, yet the offer of support for weight loss is not well established in primary care. There are numerous explanations for this, including lack of confidence in offering weight management support, a lack of locally-commissioned services or in the efficacy of the services that do exist and concerns about weight stigma.^13^ Accessibility of these specialist weight management services, known to be effective, should be improved to reduce these barriers and, over time, enable this to become part of routine care.

Despite the health benefits of weight loss, the impact of the programme on future NHS and domiciliary care costs were estimated to increase under this programme. This is because the short-term cost savings of BMI-dependent disease were smaller than age-dependent increases in diseases due to longevity, particularly for females (findings plotted in online supplemental figures 6–8). For example, back pain was modelled to increase due to the combination of being non-fatal and strongly associated with both age and BMI, so where mortality is avoided at one point, people have multiplicative risks of back pain in the added years of life. Likewise, cases of colorectal cancer also increased overall due to longevity. Other health economic analyses do not always include these additional costs accrued due to longevity as they are hard to quantify without very long trial follow-up or simulation modelling.

This intervention was more cost-effective than other more intensive programmes, including total diet replacement^12^ or drug therapies^14^ and should be considered as an early intervention. However, the average BMI of the population referred to the programme was 37 kg/m^2^ and many of these patients would benefit from a programme which leads to greater absolute weight loss with proportionally larger health gains, and/or earlier weight management support.

The intervention was found to be highly cost-effective from a health and social care perspective over the lifetime, at £4200 per QALY, falling well below the £20 000–£30 000 willingness to pay threshold guidance from NICE. These data should encourage healthcare commissioners and practitioners to make greater use of these programmes to prevent future ill-health.

Nonetheless, it is notable that more than half of the cost is attributable to the GP incentive for referrals, because only 16% of those referred complete the programme and incur the provider fee. In a parallel analysis, we have shown that the incentive was very successful in initially boosting referrals to the programme and then remained approximately stable over the next 3 years.^15^ Anecdotal reports suggest that a proportion of referrals through the NES are made via text message or similarly brief communication from the practice to patients. Further research is warranted to identify whether an in-person referral (face to face or remotely) would boost participation and/or payments could be conditional on patients enrolling in a programme. Or, if referral was implemented as part of routine care, without the incentive, it would be possible for more than twice as many patients to benefit from the programme for the same cost.

In summary, the NES incentive with referral to the Digital Weight Management Programme was highly cost effective. Around half the cost of the total system was attributable to GP payments and if referrals were an accepted part of routine primary care and part of the core service, it would enable these additional costs to be invested in expanding access to this or similar cost-effective weight management services.

## Supplementary material

10.1136/bmjopen-2026-117143 online supplemental file 1

## Data Availability

No data are available.
