# Supplementary material for: Estimating the cost-effectiveness of the National Weight Management Enhanced Service and NHS Digital Weight Management Programme in England: a modelling study
Source: BMJ Open. 2026 Oct 12;16(10):e117143. doi: 10.1136/bmjopen-2026-117143 (PMC13662030; doi:10.1136/bmjopen-2026-117143)
Supplement: Supplementary file 1 [file bmjopen-2026-117143.reviewer_comments.pdf]

PEER REVIEW HISTORY

BMJ Open publishes all reviews undertaken for accepted manuscripts. Reviewers are asked to complete a checklist review form and are provided with free text boxes to elaborate on their assessment. These free text comments are reproduced below.

ARTICLE DETAILS

Title (Provisional)

Estimating the cost-effectiveness of the National Weight Management Enhanced Service and NHS Digital Weight Management Programme in England: a modelling study

Authors

Amies-Cull, Ben; Haffner, Stella; Scarborough, Peter; Mounsey, Sarah; Aveyard, Paul; Jebb, Susan

---

VERSION 1 - REVIEW

**Reviewer**	**1**
**Name**	**Kelson, Zoe**
**Affiliation**	**University of Exeter, Mathematics**
**Date**	**23-Mar-2026**
**COI**	**none**

This study aimed to assess the longterm cost-effectiveness of the National Enhanced Service (NES) Programme.

Reviewer comments:

"Weight loss was estimated in 5-year age and sex groups for completing participants. The PRIMEtime model (a population model) was used to estimate long-term impacts of this weight loss on cases of 20 non-communicable diseases over 30 years, and on Quality-Adjusted LifeYears (QALYs), costs to the NHS and domiciliary social care over participants' lifetimes, discounted at 3.5% for health and costs." [Asbtract]

The analytical approach taken by the authors has been succinctly summarised in the Abstract.

"A total of 31,753 participants completed the Programme and lost an mean of 1.18kg/m 2 (1.15 – 1.21) for males and 1.20kg/m 2 (1.18 - 1.22) for females. Over the lifetime, 0.958 QALYs (0.0746 – 0.126) were saved per male and 0.110 (0.864 – 0.146) per female, with £65.0 (£31.8 – 111) and £170 (£112 – 248) costs to the NHS, respectively, and £50.9 (£20.7 – 72.0) and £229 (£172 – 300) to social care. Intervention costs were £133.49 per completer,

giving a cost per QALY of £4,202 (£3,437 – 5,064) from a health and social care perspective, so highly cost-effective. This was robust in probabilistic sensitivity analysis and a range of deterministic sensitivity analyses on weight regain, counterfactual weight loss and discount rates" [Asbtract]

The study outcomes are reported concisely and precisely in the Abstract, satisfactorily communicating uncertainty.

"The data were compiled by NHSE using referral information from participants' GPs and programme data from service providers."

Can the authors please comment on the completeness and coverage of this data?

"The dataset included demographics, eligibility criteria and relevant comorbidities at point of referral. Providers populated a minimum dataset that included individual participants' self-reported weight measurements and weekly engagement with the provider programme. "

Can the authors please confirm if there was any missing covariate data?

Can the potential for self-reporting bias please be acknowledged?

"Outlier BMI starting values and change values >5 SD from the mean were treated as non-valid and excluded"

Can reasons for outliers please be explored by the authors?

"Participants without two reported BMI values and outliers were assumed to have had no weight change. This approach is slightly different from that taken in Taylor et al., 2024, particularly in that those without valid weight measurements were excluded from the estimate of mean weight change in the previous paper but included as zero change here, to avoid biasing up the intervention effect while maintaining the correct number of participants who completed the intervention"

and

"Of these, 161 participants were assumed to have zero weight loss, due to not having two weight measurements or having outlying baseline BMI or BMI change values."

Can the authors please perform a sensitivity analysis here (i.e. performing a complete-case analysis)?

"PRIMEtime is a proportional multistate lifetable model (PMSL) (Barendregt et al., 1998), which simulates long-term outcomes of an exposure to a risk factor, twice in parallel: once at baseline and once with a scenario alteration to risk factor exposure applied... Here, PRIMEtime estimated the impact on the number of cases of 20 BMI-related diseases: ..."

A suitable model has been applied, which is clearly cited by the authors.

"Disease outcomes are mutually independent of each other with the exception of type-2 diabetes, as this confers additional risk for IHD and stroke. The prevalence of type-2 diabetes

in this population was much higher than the general population, so this was accounted for separately. Details of the method used to account for increased diabetes risk is described in the supplementary material."

Can more detail on this increased risk and how it was handled in the model please be included in the main article?

"UK utility decrements (Sullivan et al., 2011, given in supplementary tables S4 and S5) were used to calculate incremental QALYs"

The authors have suitably tabulated uncertainty for the utility values.

"Six sensitivity analyses were performed. .."

A number of valuable and rigorous sensitivity analyses have been explored.

"These 31,753 participants make up this study's sample for further analyses and health modelling, and further baseline and demographic data are given in Table 1"

Can the authors please further comment on whether the included cohort can be considered to be representative?

The Results are reported accurately, with uncertainty suitably communicated.

"Figure 1: 5-year age and sex group average body weight changes achieved in the NHS Digital Weight Management Programme (points) with smoothed average (lines, by GAM regression). "

Can the authors please check why the Female GAM line plotted here is linear rather than smoothed (as for the Male GAM line)?

"Table 2: Modelled impact of the NHS Digital Weight Management Programme on health and cost outcomes"

Can the authors please consider also calculating and reporting ICERs (Incremental Cost-Effectiveness Ratios)?

"It incorporates detailed cost data to allow cost-effectiveness of the Programme to be estimated, though some of these costs were commercially sensitive so not available - The study design of the effect of the Programme relies on observational data" [Strengths and Limitations]

and

"Due to this being unblinded observational data, there is risk of reporting bias due to desirability of losing more weight during an intervention. As the amount of weight loss was similar to what we might expect and sensitivity analyses having modest impacts on interpretation of programme cost-effectiveness, this is unlikely to be a major concern. PRIMEtime was developed as a population model to represent the average risks across the population of England. We were able to adapt it to account for increased baseline risks

associated with this patient group's high rates of type-2 diabetes, which were reported in the NHS England data, though we could not account for the possibility of diabetes remission. This was likely to be low given the modest weight loss and likely regain over time. Accounting for hypertension-related risk fell outside the scope of the project due to data limitations limiting the ability to appropriately capture this risk. This was due to participants being classified as a binary hypertensive/ not hypertensive rather than having a continuous blood pressure measurement in mmHg. The choice of counterfactual in the health modelling is taken to be no weight loss, though it is possible that participants who are offered a referral to a programme might be motivated to undertake increased personal efforts to manage their weight even if they do not attend the prescribed programme. We have previously shown that following opportunistic advice to recommend weight loss, 79% of people reported taking action over the following 12 weeks with a mean weight loss across the whole group of 1.18kg (Aveyard et al., 2016). The sensitivity analysis exploring this found a modest reduction the cost-effectiveness of the intervention, as would be expected, but still representing good value for money to the NHS. Wider social and economic costs were not included in this analysis, such as absenteeism, disability-related unemployment, or working age mortality. These would be likely to enhance the costeffectiveness, though there is also potential for the intervention to increase pension costs. Informal social care hours are also not included, which are likely to be a considerable social benefit, given the strength of association between BMI and informal care. (Copley et al., 2017)"

A Discussion on the study limitations is provided by the authors.

Thanks for providing a copy of the CHEERS checklist.

**Reviewer**	**2**
**Name**	**Van Der Meer, Robert B.**
**Affiliation**	**University of Strathclyde, Management Science**
**Date**	**02-Apr-2026**
**COI**	**none**

This is an interesting paper which, however, is let somewhat down by some errors in editing. My comments mainly concern the abstract and the discussion, in addition to some miscellaneous comments.

(1) Page 3, lines 21-22. Mean weight loss is expressed as kg/m2. This appears to refer to BMI – but, if so, that should be explicitly stated. (In several other instances in the paper, mean weight loss is expressed as kg.)

(2) Page 3, lines 22-23. 0.958 QALYSs should be 0.0958 QALYs.

(3) Page 3, lines 23-24. (0.864 – 0.146) should be (0.0864 – 0.146).

(4) Page 6, lines 18-19. The post-intervention weight regain at 0.33kg/year seems rather slow, although I accept the relevant reference based on a systematic review of behavioural programmes. In the present study, mean weight loss for completers is reported (page 8, lines 12-13) as 3.68 kg for males and 3.17 kg for females. This would imply that completers would tend regain their baseline weight some 9 – 11 years after the intervention. I note the deterministic sensitivity analysis of 1.5 times the rate of regain (page 11, lines 36-37). It may also be worthwhile to analyse a rate of regain of 2.0 times – although I suspect that this will not fundamentally change the overall conclusion.

(5) Table 1, pages 7-8. Potentially interesting data are provided on ethnicity (e.g., black males seem to be underrepresented compared to black females) and deprivation – but there is no further analysis of such issues in the discussion section.

(6) Page 10, lines 4-5. It seems at first surprising that incident cases of colorectal cancer have increased as a result of the weight management programme. I presume that this is principally due to increased longevity – but this may warrant a brief mention in the discussion section.

(7) Page 10, line 17. Table 2 refers to "participants". In other instances in the paper, the term "completers" is used. I presume that the people in Table 2 are completers – if so, it's best to say so explicitly (and to be fully consistent in nomenclature),

(8) Page 11, health economic evaluation. The estimated net cost per QALY is report as £4202. As this is a weighted average for the cost/QALY for males and females, it will be helpful to also report the respective cost/QALY for males and females separately. The cost/QALY for males is rather lower than for females, which seems interesting information.

(9) Page 11, line 33. On my (rather rough) calculation, the cost/QALY including social care but excluding referral costs would seem rather lower than your reported cost of £3499 (because referral costs are a substantial cost element for males) but I may be wrong on this. Nevertheless, you may wish to check.

(10) Pages 14-15, implications for policy and practice. An additional implication would be to conduct further research on how behavioural weight management programmes can best be combined with medication-based programmes (particularly, because the post-intervention weight regain in the latter appears to be much faster).

VERSION 1 - AUTHOR RESPONSE

Reviewer: 1
Prof. Zoe Kelson, University of Exeter
Comments to the Author:
This study aimed to assess the longterm cost-effectiveness of the National Enhanced Service (NES) Programme.

Reviewer comments:

"Weight loss was estimated in 5-year age and sex groups for completing participants. The PRIMEtime model (a population model) was used to estimate long-term impacts of this weight loss on cases of 20 non-communicable diseases over 30 years, and on Quality-Adjusted LifeYears (QALYs), costs to the NHS and domiciliary social care over participants' lifetimes, discounted at 3.5% for health and costs." [Asbtract]
The analytical approach taken by the authors has been succinctly summarised in the Abstract.

"A total of 31,753 participants completed the Programme and lost an mean of 1.18kg/m 2 (1.15 – 1.21) for males and 1.20kg/m 2 (1.18 - 1.22) for females. Over the lifetime, 0.958 QALYs (0.0746 – 0.126) were saved per male and 0.110 (0.864 – 0.146) per female, with £65.0 (£31.8 – 111) and £170 (£112 – 248) costs to the NHS, respectively, and £50.9 (£20.7 – 72.0) and £229 (£172 – 300) to social care. Intervention costs were £133.49 per completer, giving a cost per QALY of £4,202 (£3,437 – 5,064) from a health and social care perspective, so highly cost-effective. This was robust in probabilistic sensitivity analysis and a range of deterministic sensitivity analyses on weight regain, counterfactual weight loss and discount rates" [Asbtract]
The study outcomes are reported concisely and precisely in the Abstract, satisfactorily communicating uncertainty.

"The data were compiled by NHSE using referral information from participants' GPs and programme data from service providers."
Can the authors please comment on the completeness and coverage of this data?

Thanks for pointing out this omission that we should have commented on in the original manuscript. Data coverage was good as any gaps from the primary care record were filled by service providers to meet data requirements and payment criteria. The following has been added to the results section:

"Given the nature of the data from the NHS record and commercial provider referrals, there was very little missing data, with 65 missing from the sex variable, none from the age variable or starting BMI."

"The dataset included demographics, eligibility criteria and relevant comorbidities at point of referral. Providers populated a minimum dataset that included individual participants' self-reported weight measurements and weekly engagement with the provider programme. "
Can the authors please confirm if there was any missing covariate data?

This is a helpful clarification to add, thank you. As patients who did not meet eligibility criteria were not accepted to the programme, these were not included in the dataset. These could be for many reasons including administrative errors in the practices, with correct complete referrals sent after being returned. For the provider data, participants could either return values or not as a binary yes/ no (0/1), so weeks where there was no engagement or weight value was recorded in the dataset as non-engagement rather than missing. Therefore all the essential administrative variables had no missing values.

Can the potential for self-reporting bias please be acknowledged?

This is a key issue in the data, though the order of magnitude of weight loss was in line with previous work, so we have highlighted and qualified this risk in the Discussion as follows:

“Due to this being unblinded observational data, the risk of reporting bias cannot be excluded. However, the amount of weight loss was similar to what we might expect and sensitivity analyses show modest impacts on the interpretation of programme cost-effectiveness.”

"Outlier BMI starting values and change values >5 SD from the mean were treated as non-valid and excluded"
Can reasons for outliers please be explored by the authors?

There was a relatively small number of outliers, but these were extreme cases that could have only been misreporting, with reported weight changes or increases or decreases in weight by over 100kg in some cases. We can only speculate on these cases, for example accidentally entering an incorrect start or end weight. In method we specify:

“This was performed as a small number of cases reported extreme values that were not credible in a 12-week weight intervention, such as reporting over 100kg of weight loss (n = 24) or even 100kg weight gain (n = 11) that were most likely due to user error.”

"Participants without two reported BMI values and outliers were assumed to have had no weight change. This approach is slightly different from that taken in Taylor et al., 2024, particularly in that those without valid weight measurements were excluded from the estimate of mean weight change in the previous paper but included as zero change here, to avoid biasing up the intervention effect while maintaining the correct number of participants who completed the intervention"
and
"Of these, 161 participants were assumed to have zero weight loss, due to not having two weight measurements or having outlying baseline BMI or BMI change values."

Can the authors please perform a sensitivity analysis here (i.e. performing a complete-case analysis)?

The 161 individuals excluded from the analysis represent <0.4% of cases so their exclusion has minimal impact on the results. However we have included this additional sensitivity analysis in the supplementary material:

It reads (with the other two added sensitivity analyses):

“Supplementary sensitivity analyses:

As a complete case analysis excluding the 161 participants without complete data, the cost/QALY was £4,223 (95% Uncertainty Interval £3,455 – 5,090).

Replacing the counterfactual case with an assumption of a 2kg weight loss rather than 1kg gives a cost/ QALY of £4,997 (£4,347 – 5,055)

Testing the weight regain rate further with a doubling of that of the primary analysis gives a cost/ QALY of £5132 (£4,792 – 6,516).”

"PRIMEtime is a proportional multistate lifetable model (PMSL) (Barendregt et al., 1998), which simulates long-term outcomes of an exposure to a risk factor, twice in parallel: once at baseline and once with a scenario alteration to risk factor exposure applied... Here, PRIMEtime estimated the impact on the number of cases of 20 BMI-related diseases: ..."
A suitable model has been applied, which is clearly cited by the authors.

"Disease outcomes are mutually independent of each other with the exception of type-2 diabetes, as this confers additional risk for IHD and stroke. The prevalence of type-2 diabetes in this population was much higher than the general population, so this was accounted for separately. Details of the method used to account for increased diabetes risk is described in the supplementary material."
Can more detail on this increased risk and how it was handled in the model please be included in the main article?

Thank you for this, we debated how much technical detail was appropriate in the main text and supplementary material, and we are sorry the main methods were too vague. Following immediately after the section you have quoted, we have added:

“In short, this relied on a separate run of the Disbayes model (Bayesian epidemiological model) to generate coherent incidence, prevalence and mortality estimates for T2DM in our population. For this run, we used prevalence estimates from the NHS England data on our cohort. The incidence and mortality rates were estimated in a separate version of

PRIMEtime, which was set up using data from the general population and which estimated the T2DM incidence and mortality impact of an increase in BMI to match the distribution seen in our NHS England cohort."

"UK utility decrements (Sullivan et al., 2011, given in supplementary tables S4 and S5) were used to calculate incremental QALYs"
The authors have suitably tabulated uncertainty for the utility values.

"Six sensitivity analyses were performed. .."
A number of valuable and rigorous sensitivity analyses have been explored.

"These 31,753 participants make up this study's sample for further analyses and health modelling, and further baseline and demographic data are given in Table 1"
Can the authors please further comment on whether the included cohort can be considered to be representative?

There are two perspectives we think are important to take into account on this question. The first is on national representativeness, which the sample certainly was not, over-representing on females (in common with most weight management interventions), older people and more deprived areas. However the more important consideration is whether it is representative of the population referred to this service. We believe it is since this was a nationally commissioned service in which providers were contractually obliged to provide information.

The Results are reported accurately, with uncertainty suitably communicated.

"Figure 1: 5-year age and sex group average body weight changes achieved in the NHS Digital Weight Management Programme (points) with smoothed average (lines, by GAM regression). "
Can the authors please check why the Female GAM line plotted here is linear rather than smoothed (as for the Male GAM line)?

Thank you for pointing this out – we apologise it was incorrectly specified and has been fixed, as below.

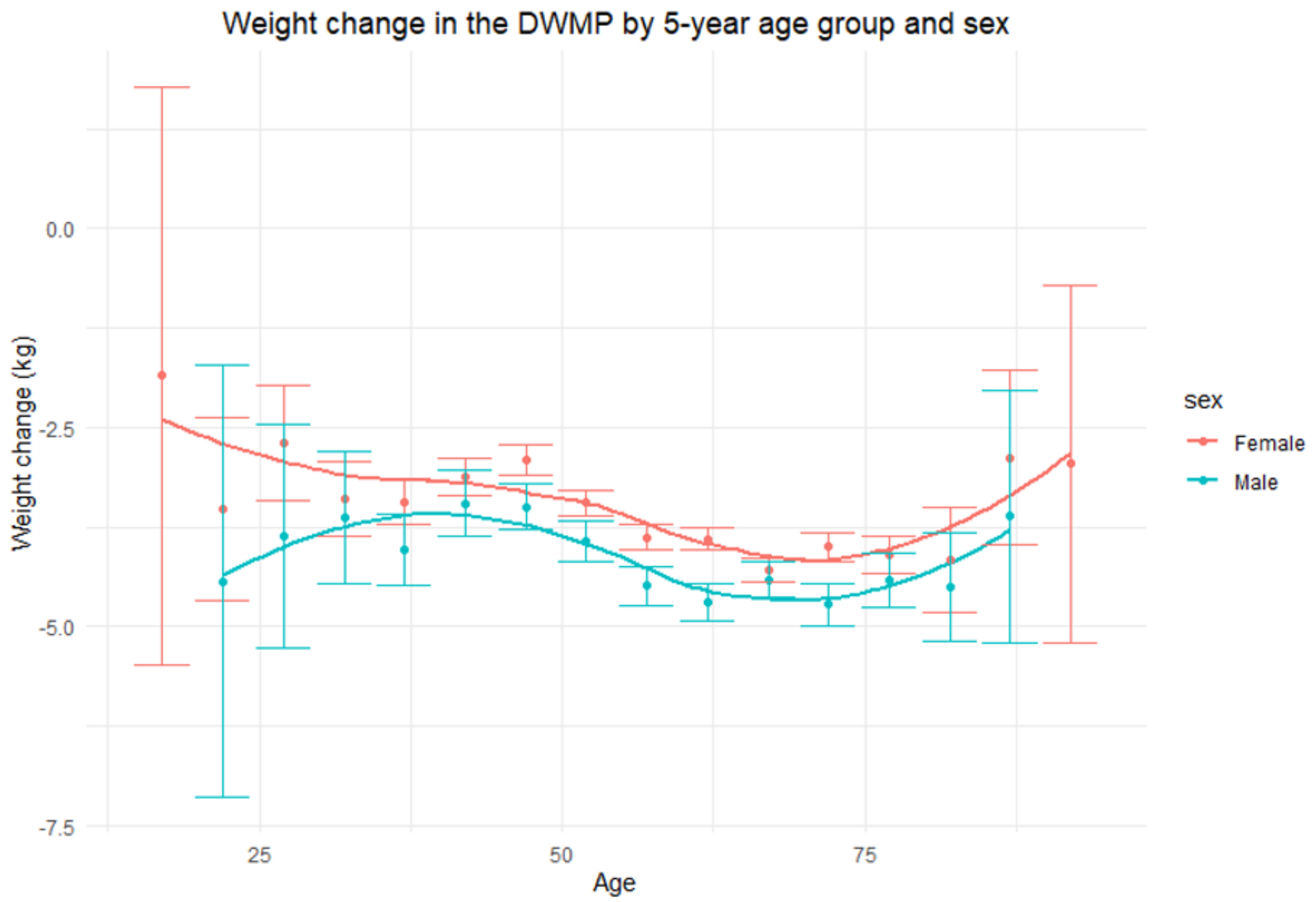

"Table 2: Modelled impact of the NHS Digital Weight Management Programme on health and cost outcomes"
Can the authors please consider also calculating and reporting ICERs (Incremental Cost-Effectiveness Ratios)?

Thank you. We did consider reporting these separately for males and females, but considered that the ICER is only interpretable in the context of a whole intervention and that the intervention would not be commissioned for only males or only females, this could be distracting. Given also that due to increased costs of longevity, it may be that the intervention was less cost effective for females, despite greater baseline risk and intervention weight loss. Therefore for equity reasons we would prefer not to report these separately.

"It incorporates detailed cost data to allow cost-effectiveness of the Programme to be estimated, though some of these costs were commercially sensitive so not available - The study design of the effect of the Programme relies on observational data" [Strengths and Limitations]
and
"Due to this being unblinded observational data, there is risk of reporting bias due to desirability of losing more weight during an intervention. As the amount of weight loss

was similar to what we might expect and sensitivity analyses having modest impacts on interpretation of programme cost-effectiveness, this is unlikely to be a major concern. PRIMEtime was developed as a population model to represent the average risks across the population of England. We were able to adapt it to account for increased baseline risks associated with this patient group's high rates of type-2 diabetes, which were reported in the NHS England data, though we could not account for the possibility of diabetes remission. This was likely to be low given the modest weight loss and likely regain over time. Accounting for hypertension-related risk fell outside the scope of the project due to data limitations limiting the ability to appropriately capture this risk. This was due to participants being classified as a binary hypertensive/ not hypertensive rather than having a continuous blood pressure measurement in mmHg. The choice of counterfactual in the health modelling is taken to be no weight loss, though it is possible that participants who are offered a referral to a programme might be motivated to undertake increased personal efforts to manage their weight even if they do not attend the prescribed programme. We have previously shown that following opportunistic advice to recommend weight loss, 79% of people reported taking action over the following 12 weeks with a mean weight loss across the whole group of 1.18kg (Aveyard et al., 2016). The sensitivity analysis exploring this found a modest reduction the cost-effectiveness of the intervention, as would be expected, but still representing good value for money to the NHS. Wider social and economic costs were not included in this analysis, such as absenteeism, disability-related unemployment, or working age mortality. These would be likely to enhance the cost-effectiveness, though there is also potential for the intervention to increase pension costs. Informal social care hours are also not included, which are likely to be a considerable social benefit, given the strength of association between BMI and informal care. (Copley et al., 2017)"
A Discussion on the study limitations is provided by the authors.

Thanks for providing a copy of the CHEERS checklist.

Thank you for your general support and helpful comments on our manuscript.

Reviewer: 2
Prof. Robert B. Van Der Meer, University of Strathclyde
Comments to the Author:
This is an interesting paper which, however, is let somewhat down by some errors in editing. My comments mainly concern the abstract and the discussion, in addition to some miscellaneous comments.
(1) Page 3, lines 21-22. Mean weight loss is expressed as kg/m2. This appears to refer to

BMI – but, if so, that should be explicitly stated. (In several other instances in the paper, mean weight loss is expressed as kg.)

Thanks for this observation, which is completely fair and we have resolved across the manuscript. Where we refer to weight change this is now expressed as kg, otherwise it is specified as BMI change in kg/m2.

(2) Page 3, lines 22-23. 0.958 QALYSs should be 0.0958 QALYs.(3) Page 3, lines 23-24. (0.864 – 0.146) should be (0.0864 – 0.146).

Thank you for identifying these errors, they have been corrected.

(4) Page 6, lines 18-19. The post-intervention weight regain at 0.33kg/year seems rather slow, although I accept the relevant reference based on a systematic review of behavioural programmes. In the present study, mean weight loss for completers is reported (page 8, lines 12-13) as 3.68 kg for males and 3.17 kg for females. This would imply that completers would tend regain their baseline weight some 9 – 11 years after the intervention. I note the deterministic sensitivity analysis of 1.5 times the rate of regain (page 11, lines 36-37). It may also be worthwhile to analyse a rate of regain of 2.0 times – although I suspect that this will not fundamentally change the overall conclusion.

We have included this as part of the supplementary material.

It reads (with the other two added sensitivity analyses):

“Supplementary sensitivity analyses:

As a complete case analysis excluding the 161 participants without complete data, the cost/QALY was £4,223 (95% Uncertainty Interval £3,455 – 5,090).

Replacing the counterfactual case with an assumption of a 2kg weight loss rather than 1kg gives a cost/ QALY of £4,997 (£4,347 – 5,055)

Testing the weight regain rate further with a doubling of that of the primary analysis gives a cost/ QALY of £5132 (£4,792 – 6,516).”

(5) Table 1, pages 7-8. Potentially interesting data are provided on ethnicity (e.g., black males seem to be underrepresented compared to black females) and deprivation – but there is no further analysis of such issues in the discussion section.

This detail is covered in a previous paper reporting the early outcomes from the programme. so we have not commented further on it here. (Taylor K, Indulkar T,

Thompson B, Pinkard C, Barron E, Frost T, et al. Early outcomes of referrals to the English National Health Service Digital Weight Management Programme. Obesity (Silver Spring). 2024 Jun 1;32(6):1083–92. doi:10.1002/OBY.24024 PubMed PMID: 38644161)

(6) Page 10, lines 4-5. It seems at first surprising that incident cases of colorectal cancer have increased as a result of the weight management programme. I presume that this is principally due to increased longevity – but this may warrant a brief mention in the discussion section.

This is exactly the explanation, which has been added to the discussion, following on from the comment about this issue on back pain:

"For example, back pain was modelled to increase due to the combination of being non-fatal and strongly associated with both age and BMI, so where mortality is avoided at one point, people have multiplicative risks of back pain in the added years of life. Likewise, cases of colorectal cancer also increased overall due to longevity."

(7) Page 10, line 17. Table 2 refers to "participants". In other instances in the paper, the term "completers" is used. I presume that the people in Table 2 are completers – if so, it's best to say so explicitly (and to be fully consistent in nomenclature),

You are entirely correct this language was not consistent and has now been changed to "completers".

(8) Page 11, health economic evaluation. The estimated net cost per QALY is report as £4202. As this is a weighted average for the cost/QALY for males and females, it will be helpful to also report the respective cost/QALY for males and females separately. The cost/QALY for males is rather lower than for females, which seems interesting information.

This is an interesting point here, thank you. The cost/QALY here is for the programme as it played out, essentially as a weighted average as you've inferred. It is likely that the intervention may be less cost effective for females due to the effects of longevity, despite greater baseline BMIs and intervention effects on BMI, but we decided not to report these in the paper. As the cost-effectiveness applies to the programme as a whole and it could not be implemented only for males or females for equity reasons, we thought this could be distracting without contributing to the interpretation of the findings.

(9) Page 11, line 33. On my (rather rough) calculation, the cost/QALY including social care but excluding referral costs would seem rather lower than your reported cost of

£3499 (because referral costs are a substantial cost element for males) but I may be wrong on this. Nevertheless, you may wish to check.

Thank you for reading this in close enough detail to pick this up. We have checked and the number is correct, though the £3,499 refers to NHS and social care costs but without referral costs. We have clarified the wording to make sure this is clear, now reading in context:

"Cost per QALY for NHS costs alone (excluding social care) was £2,596 (95% UI £2,088 – £3,138). Including both NHS and social care costs but excluding referral costs, it would be £3,499 (£2,854 – £4,233)."

(10) Pages 14-15, implications for policy and practice. An additional implication would be to conduct further research on how behavioural weight management programmes can best be combined with medication-based programmes (particularly, because the post-intervention weight regain in the latter appears to be much faster).

We recognise the landscape for weight management is changing fast. However we do not think the comparison with new weight-loss medication can be easily addressed here since the eligible population and the expected outcomes vary substantially. It is beyond the scope of this cost-effectiveness analysis to speculate on future combinations of therapies.

Thank you for your helpful comments on the content and presentation of our manuscript and we hope these changes have meaningfully improved its quality for publication.

VERSION 2 - REVIEW

**Reviewer**	**1**
**Name**	**Kelson, Zoe**
**Affiliation**	**University of Exeter, Mathematics**
**Date**	**04-Aug-2026**
**COI**	

Thanks to the authors for their responses, clarifications, additional analyses, and revisions, which have strengthened the reporting of this study.
