# Supplementary material for: Estimating the cost-effectiveness of the National Weight Management Enhanced Service and NHS Digital Weight Management Programme in England: a modelling study
Source: BMJ Open. 2026 Oct 12;16(10):e117143. doi: 10.1136/bmjopen-2026-117143 (PMC13662030; doi:10.1136/bmjopen-2026-117143)
Supplement: Supplementary file 2 [file bmjopen-16-10-s001.docx]

# Supplementary material

## Supplementary figure 1

Supplementary figure 1: Diagram of patient flow through the treatment pathway of the Digital Weight Management Programme.

## Supplementary methods for type-2 diabetes epidemiology

The study participants had greater T2DM prevalence than the general population, which needed to be accounted for in the PRIMEtime modelling. This is more fully explained in the supplementary material, but briefly, type-2 diabetes prevalence was taken from the study population, with the underlying population structure of the sample (population pyramid), incidence and mortality rates estimated in a specifically-prepared version of PRIMEtime. These parameters were input into the Disbayes model, (1,2)designed specifically for PMSLs to estimate consistent sets of epidemiological model inputs and estimate case fatality rates. Relative Risks for this are listed below in table S1.

Population-specific type-2 diabetes incidence and mortality rates were estimated in a separate specifically-prepared version of PRIMEtime. This involved setting the baseline BMIs for the population as those observed in the general population by age and sex (from Health Survey for England 2018 and smoothed over age groups) and setting the scenario BMIs to those of the participants at the start of the DWMP. PRIMEtime then calculated the Population Impact Fractions (PIFs) on T2DM incidence for these difference in BMI, and in turn the disease incidence of T2DM expected for the study population.

T2DM mortality rates were calculated similarly within this separate version of PRIMEtime, by calculating a PIF for T2DM-specific mortality due to BMI, based on relative risks from a prospective cohort study.(3) This PIF was then applied to T2DM mortality rates of the general population calculated within PRIMEtime, to give estimated T2DM mortality rates for the study population.

Finally, all-cause mortality rates calculated in the same way as for T2DM-specific mortality, using the relative risk for all-cause mortality from the same (3) prospective cohort study analysis. These were applied to a population of 1,000 males and 1,000 females from age 20 (when the prospective cohort study population starts) and used to generate a synthetic population structure for the study population that underlies the sample in the programme. These prevalence, incidence and mortality rates, and population structure were entered into the Disbayes model, (1,2) which is a Bayesian epidemiological model designed to estimate internally-consistent sets of disease epidemiology from different sources and to estimate case fatality rates, specifically for use in multistate lifetables such as PRIMEtime.

### Table S1: Diabetes relative risks for estimating baseline type-2 diabetes epidemiology

| Type-2 diabetes mortality | | |  |
| --- | --- | --- | --- |
| BMI range | Mean | Lower CI | Upper CI |
| 0 - 18.5 | 1.01 | 0.68 | 1.52 |
| 18.5 - 20 | 1.01 | 0.74 | 1.38 |
| 20 - 22.5 | 0.91 | 0.76 | 1.09 |
| 22.5 - 25 | 1 | 1 | 1 |
| 25 - 27.5 | 1.18 | 1.03 | 1.35 |
| 27.5 - 30 | 1.09 | 0.93 | 1.26 |
| 30 - 35 | 1.58 | 1.38 | 1.82 |
| 35 - 40 | 2.6 | 2.18 | 3.11 |
| 40 - 99 | 3.58 | 2.83 | 4.53 |
|  |  |  |  |
| All-cause mortality | |  |  |
| BMI range | Mean | Lower CI | Upper CI |
| 0 - 18.5 | 1.56 | 1.52 | 1.59 |
| 18.5 - 20 | 1.29 | 1.27 | 1.32 |
| 20 - 22.5 | 1.11 | 1.1 | 1.12 |
| 22.5 - 25 | 1 | 1 | 1 |
| 25 - 27.5 | 0.98 | 0.97 | 0.99 |
| 27.5 - 30 | 1.01 | 1 | 1.03 |
| 30 - 35 | 1.12 | 1.1 | 1.13 |
| 35 - 40 | 1.36 | 1.33 | 1.38 |
| 40 - 99 | 1.88 | 1.83 | 1.93 |

From (3)

## PRIMEtime input parameters

Supplementary figure 1: Baseline disease incidence rates

Supplementary figure 2: baseline disease prevalence rates

Supplementary figure 3: baseline disease case fatality rates

Supplementary figure 4: epidemiology for acute diseases

Supplementary figure 5: epidemiology for non-fatal conditions

### Table S2: BMI-disease outcome Relative Risks

|  | | BMI units | Subgroup | Mean | CI lower | CI upper | Reference |
| --- | --- | --- | --- | --- | --- | --- | --- |
| All-cause mortality | | 15 to <18.5 | Males | 1.83 | 1.66 | 2.02 | (4) |
|  |  | 18.5 to <25 | Males | 1 | 0.98 | 1.02 |  |
|  |  | 25 to <30 | Males | 1.12 | 1.11 | 1.13 |  |
|  |  | 30 to <35 | Males | 1.7 | 1.63 | 1.77 |  |
|  |  | 35 to <40 | Males | 2.68 | 2.54 | 2.83 |  |
|  |  | 40 to <60 | Males | 4.24 | 3.77 | 4.76 |  |
|  |  | 15 to <18.5 | Females | 1.53 | 1.44 | 1.62 |  |
|  |  | 18.5 to <25 | Females | 1 | 0.98 | 1.02 |  |
|  |  | 25 to <30 | Females | 1.08 | 1.07 | 1.1 |  |
|  |  | 30 to <35 | Females | 1.37 | 1.34 | 1.4 |  |
|  |  | 35 to <40 | Females | 1.86 | 1.79 | 1.93 |  |
|  |  | 40 to <60 | Females | 2.73 | 2.57 | 2.91 |  |
| Ischemic heart disease | | Per 5kg/m^2^ | 35-44 | 1.66 | 1.51 | 1.84 | (5) |
|  |  | Per 5kg/m^2^ | 45-54 | 1.55 | 1.46 | 1.64 |  |
|  |  | Per 5kg/m^2^ | 55-64 | 1.44 | 1.4 | 1.48 |  |
|  |  | Per 5kg/m^2^ | 65-74 | 1.35 | 1.32 | 1.38 |  |
|  |  | Per 5kg/m^2^ | 75-84 | 1.26 | 1.2 | 1.32 |  |
|  |  | Per 5kg/m^2^ | 85+ | 1.14 | 1.04 | 1.26 |  |
| Ischemic stroke |  | Per 5kg/m^2^ | 35-44 | 1.86 | 1.67 | 2.08 | (5) |
|  |  | Per 5kg/m^2^ | 45-54 | 1.67 | 1.53 | 1.81 |  |
|  |  | Per 5kg/m^2^ | 55-64 | 1.5 | 1.4 | 1.6 |  |
|  |  | Per 5kg/m^2^ | 65-74 | 1.35 | 1.28 | 1.41 |  |
|  |  | Per 5kg/m^2^ | 75-84 | 1.21 | 1.16 | 1.26 |  |
|  |  | Per 5kg/m^2^ | 85+ | 1.04 | 0.96 | 1.12 |  |
| Intracerebral hemorrhage | | Per 5kg/m^2^ | 35-44 | 2.54 | 1.96 | 3.28 | (5) |
|  |  | Per 5kg/m^2^ | 45-54 | 2.1 | 1.66 | 2.66 |  |
|  |  | Per 5kg/m^2^ | 55-64 | 1.75 | 1.44 | 2.13 |  |
|  |  | Per 5kg/m^2^ | 65-74 | 1.48 | 1.29 | 1.71 |  |
|  |  | Per 5kg/m^2^ | 75-84 | 1.3 | 1.21 | 1.4 |  |
|  |  | Per 5kg/m^2^ | 85+ | 1.05 | 0.92 | 1.2 |  |
| Hypertensive heart disease | | Per 5kg/m^2^ | 35-44 | 2.15 | 0.8 | 5.78 | (5) |
|  |  | Per 5kg/m^2^ | 45-54 | 2.02 | 0.97 | 4.21 |  |
|  |  | Per 5kg/m^2^ | 55-64 | 1.9 | 1.17 | 3.07 |  |
|  |  | Per 5kg/m^2^ | 65-74 | 1.81 | 1.45 | 2.26 |  |
|  |  | Per 5kg/m^2^ | 75-84 | 1.63 | 1.53 | 1.74 |  |
|  |  | Per 5kg/m^2^ | 85+ | 1.45 | 1.05 | 2.01 |  |
| Diabetes mellitus type 2 | | Per 5kg/m^2^ | 35-44 | 3.07 | 2.28 | 4.15 | (5) |
|  |  | Per 5kg/m^2^ | 45-54 | 2.66 | 2.15 | 3.3 |  |
|  |  | Per 5kg/m^2^ | 55-64 | 2.32 | 2.04 | 2.63 |  |
|  |  | Per 5kg/m^2^ | 65-74 | 2.03 | 1.95 | 2.11 |  |
|  |  | Per 5kg/m^2^ | 75-84 | 1.7 | 1.61 | 1.79 |  |
|  |  | Per 5kg/m^2^ | 85+ | 1.38 | 1.23 | 1.56 |  |
| Atrial fibrillation and flutter | | Per 5kg/m^2^ |  | 1.28 | 1.2 | 1.38 | (6) |
| Gallbladder and biliary diseases | | Per 5kg/m^2^ |  | 1.63 | 1.49 | 1.78 | (7) |
| Colon and rectum cancer | | Per 5kg/m^2^ |  | 1.05 | 1.03 | 1.07 | (8) |
| Breast cancer |  | Per 5kg/m^2^ |  | 1.12 | 1.09 | 1.15 |  |
| Uterine cancer |  | Per 5kg/m^2^ |  | 1.54 | 1.47 | 1.61 | (9) |
| Esophageal cancer* | | Per 5kg/m^2^ | adenocarcinoma | 1.54 | 1.41 | 1.67 | (9) |
|  |  | Per 5kg/m^2^ | squamous cell carcinoma | 0.63 | 0.53 | 0.75 | (9) |
| Kidney cancer |  | Per 5kg/m^2^ |  | 1.24 | 1.17 | 1.32 | (9) |
|  |  | Per 5kg/m^2^ |  | 1.33 | 1.25 | 1.42 | (9) |
| Pancreatic cancer | | Per 5kg/m^2^ |  | 1.1 | 1.06 | 1.14 | (9) |
| Multiple myeloma | | Per 5kg/m^2^ |  | 1.12 | 1.09 | 1.15 | (9) |
| Liver cancer |  | Per 5kg/m^2^ |  | 1.3 | 1.16 | 1.46 | (8) |
| Asthma |  | healthy | children | 1 | 1 | 1 | (10) |
|  |  | overweight | children | 1.64 | 1.13 | 2.38 |  |
|  |  | obese | children | 1.92 | 1.39 | 2.65 |  |
|  |  | <25 | adults | 1 | 1 | 1 | (11) |
|  |  | 25-29.9 | adults | 1.38 | 1.17 | 1.62 |  |
|  |  | 30+ | adults | 1.92 | 1.43 | 2.59 |  |
| Low back pain |  | <25 |  | 1 | 1 | 1 | (12) |
|  |  | 25-29.9 |  | 1.08 | 0.9 | 1.29 |  |
|  |  | 30+ |  | 1.42 | 1.11 | 0.181 |  |
| Osteoarthritis knee | | <25 | 50+ | 1 | 1 | 1 | (13) |
|  |  | 25-30 |  | 1.98 | 1.57 | 2.2 |  |
|  |  | >30 |  | 2.66 | 2.15 | 3.28 |  |
| Osteoarthritis hip | | Per 5kg/m^2^ |  | 1.11 | 1.07 | 1.16 | (14) |
| Depressive disorders | | <25 |  | 1 | 1 | 1 | (15) |
|  |  | 25-29.9 |  | 1.04 | 0.99 | 1.11 |  |
|  |  | 30+ |  | 1.15 | 1.06 | 1.25 |  |

### Table S3: Diabetes-disease outcome Relative Risks

| Condition | Subgroup | | Mean | Lower CI | Upper CI |  |
| --- | --- | --- | --- | --- | --- | --- |
| Ischemic heart disease | Males | | 1.85 | 1.64 | 2.1 | (16) |
| Ischemic heart disease | Females | | 2.63 | 2.27 | 3.06 |  |
| Ischemic stroke |  | Males | 1.83 | 1.6 | 2.08 | (17) |
| Ischemic stroke |  | Females | 2.28 | 1.93 | 2.69 |  |

### Table S4: Disease state utility decrements

| Condition | Mean | CI lower | CI upper |
| --- | --- | --- | --- |
| Ischemic heart disease incidence | -0.06257 | -0.08839 | -0.03676 |
| Ischemic heart disease prevalence | -0.0368 | -0.08724 | 0.013645 |
| Ischemic stroke incidence | -0.11705 | -0.14085 | -0.09325 |
| Ischemic stroke prevalence | -0.0732 | -0.12096 | -0.02543 |
| Intracerebral hemorrhage incidence | -0.11705 | -0.14085 | -0.09325 |
| Intracerebral hemorrhage prevalence | -0.0732 | -0.12096 | -0.02543 |
| Hypertensive heart disease | -0.04604 | -0.05422 | -0.03786 |
| Diabetes mellitus type 2 | -0.07143 | -0.08093 | -0.06194 |
| Atrial fibrillation and flutter | -0.03839 | -0.05184 | -0.02494 |
| Colon and rectum cancer | -0.06739 | -0.10108 | -0.0337 |
| Breast cancer | -0.01943 | -0.04766 | 0.008801 |
| Uterine cancer | -0.00993 | -0.06166 | 0.041802 |
| Esophageal cancer | -0.00993 | -0.06166 | 0.041802 |
| Kidney cancer | -0.048 | -0.12914 | 0.033146 |
| Pancreatic cancer | -0.00993 | -0.06166 | 0.041802 |
| Multiple myeloma | -0.08589 | -0.13882 | -0.03296 |
| Liver cancer | -0.09295 | -0.17871 | -0.00718 |
| Asthma | -0.04634 | -0.05889 | -0.03379 |
| Low back pain | -0.0866 | -0.09742 | -0.07577 |
| Osteoarthritis hip | -0.11445 | -0.12929 | -0.09961 |
| Osteoarthritis knee | -0.11445 | -0.12929 | -0.09961 |
| Depressive disorders | -0.12691 | -0.14717 | -0.10665 |
| Dental caries | -0.0021 | -0.02615 | 0.02194 |
| Gallbladder and biliary diseases* | -0.06211 | -0.06211 | -0.06211 |
| *weighted average based on HSE admissions data (18)  (19) | | | |

### Table S5: Baseline average utility values

| State | Mean | CI lower | CI upper |
| --- | --- | --- | --- |
| Age (continuous years) | -0.00027 | -0.0006 | 4.87E-05 |
| Male | 0.001005 | -0.00023 | 0.002236 |
| Age 10-19 | 0.913 | 0.90418 | 0.92182 |
| Age 20-29 | 0.905 | 0.900884 | 0.909116 |
| Age 30-39 | 0.879 | 0.874884 | 0.883116 |
| Age 40-49 | 0.837 | 0.831512 | 0.842488 |
| Age 50-59 | 0.798 | 0.79114 | 0.80486 |
| Age 60-69 | 0.774 | 0.766356 | 0.781644 |
| Age 70-79 | 0.723 | 0.713396 | 0.732604 |
| Age 80-89 | 0.657 | 0.6423 | 0.6717 |
| BMI <=18 years |  |  |  |
| Healthy | 0.85 | 0.84 | 0.87 |
| Overweight | 0.83 | 0.81 | 0.85 |
| Obese  (19) | 0.82 | 0.79 | 0.84 |

### Table S6: Healthcare costs

| Condition | Unit (£, 2018) | Mean | CI lower | CI upper |
| --- | --- | --- | --- | --- |
| Ischemic heart disease | £/prevalent case | 606.3221 | 368.6439 | 844.0004 |
| Ischemic stroke | £/prevalent case | 1949.97 | 1185.582 | 2714.359 |
| Intracerebral hemorrhage | £/prevalent case | 2563.242 | 1558.451 | 3568.033 |
| Hypertensive heart disease | £/prevalent case | 103.4775 | 62.91433 | 144.0407 |
| Diabetes mellitus type 2 | £/prevalent case | 186.6225 | 113.4665 | 259.7785 |
| Atrial fibrillation and flutter | £/prevalent case | 195.2957 | 118.7398 | 271.8516 |
| Colon and rectum cancer | £/incident case | 9204.137 | 5596.116 | 12812.16 |
| Breast cancer | £/incident case | 12432.79 | 7559.134 | 17306.44 |
| Uterine cancer | £/incident case | 2059.513 | 1252.184 | 2866.842 |
| Esophageal cancer | £/incident case | 2420.799 | 1471.846 | 3369.753 |
| Kidney cancer | £/incident case | 4979.266 | 3027.394 | 6931.138 |
| Pancreatic cancer | £/incident case | 2695.389 | 1638.796 | 3751.981 |
| Multiple myeloma | £/incident case | 22915.1 | 13932.38 | 31897.82 |
| Liver cancer | £/incident case | 2171.54 | 1320.297 | 3022.784 |
| Asthma | £/incident case | 4186.372 | 2545.314 | 5827.43 |
| Low back pain | £/incident case | 425.1382 | 258.484 | 591.7924 |
| Osteoarthritis hip | £/incident case | 13951.01 | 8482.217 | 19419.81 |
| Osteoarthritis knee | £/incident case | 1799.225 | 1093.929 | 2504.521 |
| Depressive disorders | £/incident case | 410.464 | 249.5621 | 571.3659 |
| Gallbladder and biliary diseases | £/incident case | 371.7499 | 226.0239 | 517.4758 |
| Total non-modelled diseases  (18) | £/person | 1099.033 | 668.2119 | 1529.854 |

## Table S7: Population numbers

| Age group | Males | Females |
| --- | --- | --- |
| 15-19 | 0 | 1 |
| 20-24 | 38 | 94 |
| 25-29 | 116 | 286 |
| 30-34 | 307 | 751 |
| 35-39 | 711 | 1388 |
| 40-44 | 903 | 1779 |
| 45-49 | 1248 | 2180 |
| 50-54 | 1586 | 2928 |
| 55-59 | 1682 | 3265 |
| 60-64 | 1693 | 3059 |
| 65-69 | 1471 | 2368 |
| 70-74 | 952 | 1425 |
| 75-79 | 539 | 707 |
| 80-84 | 112 | 119 |
| 85-89 | 0 | 23 |

## Table S8: Baseline BMI values (smoothed, crude mean by 5 year groups)

| Sex | Age | Mean BMI | SD BMI | Mean height (m) |
| --- | --- | --- | --- | --- |
| male | 18-24 | 38.90417 | 7.448042 | 1.774528 |
| male | 25-29 | 38.54441 | 7.302535 | 1.774686 |
| male | 30-34 | 38.22662 | 7.138667 | 1.77417 |
| male | 35-39 | 37.89295 | 6.937199 | 1.773081 |
| male | 40-44 | 37.54341 | 6.698132 | 1.771421 |
| male | 45-49 | 37.17799 | 6.421465 | 1.76919 |
| male | 50-54 | 36.79671 | 6.107199 | 1.766386 |
| male | 55-59 | 36.39954 | 5.755333 | 1.76301 |
| male | 60-64 | 35.9865 | 5.365867 | 1.759063 |
| male | 65-69 | 35.55758 | 4.938802 | 1.754544 |
| male | 70-74 | 35.1128 | 4.474138 | 1.749453 |
| male | 75-79 | 34.65213 | 3.971874 | 1.74379 |
| male | 80-84 | 34.1756 | 3.432011 | 1.737555 |
| female | 18-24 | 40.68346 | 7.889636 | 1.645989 |
| female | 25-29 | 40.34334 | 7.812826 | 1.644474 |
| female | 30-34 | 39.98734 | 7.698416 | 1.642387 |
| female | 35-39 | 39.61546 | 7.546407 | 1.639729 |
| female | 40-44 | 39.22772 | 7.356798 | 1.636498 |
| female | 45-49 | 38.82409 | 7.129589 | 1.632696 |
| female | 50-54 | 38.4046 | 6.864782 | 1.628322 |
| female | 55-59 | 37.96922 | 6.562374 | 1.623376 |
| female | 60-64 | 37.51798 | 6.222367 | 1.617858 |
| female | 65-69 | 37.05086 | 5.844761 | 1.611768 |
| female | 70-74 | 36.56786 | 5.429555 | 1.605107 |
| female | 75-79 | 36.06899 | 4.976749 | 1.597873 |
| female | 80-84 | 35.55425 | 4.486344 | 1.590068 |
| female | 85-89 | 35.02362 | 3.95834 | 1.581691 |

## Supplementary sensitivity analyses

As a complete case analysis excluding the 161 participants without complete data, the cost/QALY was £4,223 (95% Uncertainty Interval £3,455 – 5,090).

Replacing the counterfactual case with an assumption of a 2kg weight loss rather than 1kg gives a cost/ QALY of £4,997 (£4,347 – 5,055)

Testing the weight regain rate further with a doubling of that of the primary analysis gives a cost/ QALY of £5132 (£4,792 – 6,516).

## Age- and time-horizon-dependent impacts on modelled costs

Supplementary figure 6

Supplementary figure 7

Supplementary figure 8

Interpretation of supplementary figures 6-8:

Supplementary figures 6-8 show the breakdown of NHS and social care costs being derived over time in the health modelling. These give starting age along the x-axis (in 5-year age groups) and undiscounted cost changes per person in £ on the y-axis. Important to interpretation, age along the x-axis represents the starting ages of different cohorts of participants, not participants ageing over time. Costs are cumulative, not accrued in individual time windows, for example, on the orange line at 20 years includes cost impacts between 0-10 years in red, plus those between 10-20 years. Take 6 and the red line representing a 10 year time horizon, this indicates that at 10 years, a small incremental cost to the NHS is incurred for age groups up to 50 years old, then a small saving is made after the age of 50. The orange, pale green and other lines in turn indicate that by 20, 30 years and so on, costs incurred are greater, with smaller cost savings to offset them. 7 shows the same but for social care with savings for older age groups at any horizon (ceasing to change once 100% mortality is applied at age 100). Extremely small changes are modelled for the age groups 20-24 years and 25-29 years as the first effects on mortality of fatal diseases is modelled to begin from age 35, so longevity effects are absent or weak in these groups during the period of time before weight is fully regained. Figure 8 shows impacts on incremental NHS and social care costs combined.

These effects are driven by two factors. First, the prevention of potentially fatal diseases results in more life years lived and greater costs, and second, that the combination of weight loss on age-related risk offsets this effect for those in the higher age groups. In both NHS and social care costs, the increase in life-years quickly offsets the decrease in BMI-related burden as weight is regained after only 12 years, leaving the remaining lifecourse to accrue more costs on health and social care. A high-risk population with raised BMI in mid-age incurs little baseline costs to incur, while a high-risk population in older age incurs a lot of costs at baseline that are multiplied in this case due to increased longevity. As these figures show undiscounted costs, this offsetting effect on younger cohorts is much greater than in the main analysis.

## References

1.	Jackson C. GitHub [Internet]. 2023 [cited 2023 Feb 22]. Bayesian estimation of chronic disease epidemiology from incomplete data: the disbayes package. Available from: https://chjackson.github.io/disbayes/articles/disbayes.html

2.	Jackson C, Zapata-Diomedi B, Woodcock J. Bayesian multistate modelling of incomplete chronic disease burden data. ArXiv. 2021 Nov 28;arXiv:2111.14100. doi:10.48550/ARXIV.2111.14100

3.	Bhaskaran K, dos-Santos-Silva I, Leon DA, Douglas IJ, Smeeth L. Association of BMI with overall and cause-specific mortality: a population-based cohort study of 3·6 million adults in the UK. Lancet Diabetes Endocrinol. 2018 Dec;6(12):944–53. doi:10.1016/S2213-8587(18)30288-2

4.	Di Angelantonio E, Bhupathiraju SN, Wormser D, Gao P, Kaptoge S, de Gonzalez AB, et al. Body-mass index and all-cause mortality: individual-participant-data meta-analysis of 239 prospective studies in four continents. The Lancet. 2016 Aug;388(10046):776–86. doi:10.1016/S0140-6736(16)30175-1

5.	Singh GM, Danaei G, Farzadfar F, Stevens GA, Woodward M, Wormser D, et al. The Age-Specific Quantitative Effects of Metabolic Risk Factors on Cardiovascular Diseases and Diabetes: A Pooled Analysis [Internet]. doi:10.1371/journal.pone.0065174

6.	Aune D, Sen A, Schlesinger S, Norat T, Janszky I, Romundstad P, et al. Body mass index, abdominal fatness, fat mass and the risk of atrial fibrillation: a systematic review and dose–response meta-analysis of prospective studies. Eur J Epidemiol. 2017 Mar 1;32(3):181–92. doi:10.1007/S10654-017-0232-4/FIGURES/5 PubMed PMID: 28194602.

7.	Aune D, Norat T, Vatten LJ. Body mass index, abdominal fatness and the risk of gallbladder disease. Eur J Epidemiol. 2015 Sep 30;30(9):1009–19. doi:10.1007/S10654-015-0081-Y/TABLES/1 PubMed PMID: 26374741.

8.	World Cancer Research Fund. Body fatness and weight gain and the risk of cancer; WCRF Continuous Update Project [Internet]. 2018 [cited 2023 Feb 23]. Available from: https://www.wcrf.org/wp-content/uploads/2021/01/Body-fatness-and-weight-gain_0.pdf

9.	Kyrgiou M, Kalliala I, Markozannes G, Gunter MJ, Paraskevaidis E, Gabra H, et al. Adiposity and cancer at major anatomical sites: umbrella review of the literature. The BMJ. 2017;356. doi:10.1136/BMJ.J477 PubMed PMID: 28246088.

10.	Azizpour Y, Delpisheh A, Montazeri Z, Sayehmiri K, Darabi B. Effect of childhood BMI on asthma: a systematic review and meta-analysis of case-control studies [Internet]. doi:10.1186/s12887-018-1093-z

11.	Beuther DA, Sutherland ER. Overweight, Obesity, and Incident Asthma. Am J Respir Crit Care Med. 2007 Apr 1;175(7):661–6. doi:10.1164/rccm.200611-1717OC

12.	Shiri R, Karppinen J, Leino-Arjas P, Solovieva S, Viikari-Juntura E. The Association Between Obesity and Low Back Pain: A Meta-Analysis. Am J Epidemiol. 2010 Jan 15;171(2):135–54. doi:10.1093/AJE/KWP356 PubMed PMID: 20007994.

13.	Silverwood V, Blagojevic-Bucknall M, Jinks C, Jordan JL, Protheroe J, Jordan KP. Current evidence on risk factors for knee osteoarthritis in older adults: A systematic review and meta-analysis. Osteoarthritis Cartilage. 2015 Apr 1;23(4):507–15. doi:10.1016/J.JOCA.2014.11.019 PubMed PMID: 25447976.

14.	Jiang L, Tian W, Wang Y, Rong J, Bao C, Liu Y, et al. Body mass index and susceptibility to knee osteoarthritis: A systematic review and meta-analysis. Joint Bone Spine. 2012 May 1;79(3):291–7. doi:10.1016/J.JBSPIN.2011.05.015 PubMed PMID: 21803633.

15.	Amiri S, Behnezhad S, Nadinlui KB. Body Mass Index (BMI) and risk of depression in adults: A systematic review and meta-analysis of longitudinal studies. Obes Med. 2018 Dec;12:1–12. doi:10.1016/j.obmed.2018.10.001

16.	Peters SAE, Huxley RR, Woodward M. Diabetes as risk factor for incident coronary heart disease in women compared with men: A systematic review and meta-analysis of 64 cohorts including 858,507 individuals and 28,203 coronary events. Diabetologia. 2014 May 25;57(8):1542–51. doi:10.1007/S00125-014-3260-6/FIGURES/3 PubMed PMID: 24859435.

17.	Peters SAE, Huxley RR, Woodward M. Diabetes as a risk factor for stroke in women compared with men: A systematic review and meta-analysis of 64 cohorts, including 775 385 individuals and 12 539 strokes. The Lancet. 2014 Jun 7;383(9933):1973–80. doi:10.1016/S0140-6736(14)60040-4 PubMed PMID: 24613026.

18.	Cobiac LJ, Law C, Scarborough P. PRIMEtime: an epidemiological model for informing diet and obesity policy. medRxiv. 2022 May 21;2022.05.18.22275284. doi:10.1101/2022.05.18.22275284

19.	Sullivan PW, Slejko JF, Sculpher MJ, Ghushchyan V. Catalogue of EQ-5D Scores for the United Kingdom. http://dx.doi.org/101177/0272989X11401031. 2011 Mar 21;31(6):800–4. doi:10.1177/0272989X11401031 PubMed PMID: 21422468.
